# Direct Peroneal and Tibial Transcutaneous Electrical Nerve Stimulation for Improving Postural Control in European Women with Diabetic Polyneuropathy: A Randomized Controlled Trial

**DOI:** 10.3390/jcm15135000

**Published:** 2026-06-26

**Authors:** Mustafa Al-Zamil, Natalia G. Kulikova, Larisa V. Smekalkina, Natalia A. Shnayder, Natalia B. Korchazhkina, Oleg S. Vasilyev, Regina F. Nasyrova, Margarita V. Naprienko, Olga V. Khripunova, Numan Mansur

**Affiliations:** 1 Department of Physiotherapy, Faculty of Continuing Medical Education, Peoples’ Friendship University of Russia, 117198 Moscow, Russia; kulikovang777@mail.ru (N.G.K.); d-64-158@mail.ru (N.M.); 2 Department of Restorative Medicine and Neurorehabilitation, Medical Dental Institute, 127253 Moscow, Russia; smekalkinal@bk.ru; 3 Department of Sports Medicine and Medical Rehabilitation, I.M. Sechenov First Moscow State Medical University, 119991 Moscow, Russia; iaam@yandex.ru (O.S.V.); mv_naprienko@mail.ru (M.V.N.); olaw@bk.ru (O.V.K.); 4 Institute of Personalized Psychiatry and Neurology, V.M. Bekhterev National Medical Research Center for Psychiatry and Neurology, 3 Bekhterev St., 192019 St. Petersburg, Russia; regina_nmrcpn@mail.ru; 5 Shared Core Facilities “Molecular and Cell Technologies”, V.F. Voino-Yasenetsky Krasnoyarsk State Medical University, 1 Partizan Zheleznyak St., 660022 Krasnoyarsk, Russia; 6 Department of Restorative Medicine and Biomedical Technologies, Federal State Educational Institution of Higher Education, Moscow State Medical and Dental University Named after A.I. Evdokimov, Ministry of Health of Russia, 127473 Moscow, Russia; n9857678103@gmail.com

**Keywords:** posturography, transcutaneous electrical nerve stimulation, postural disorders, envelope area, electromyography, diabetic polyneuropathy

## Abstract

**Background:** Postural disability develops in almost all patients with diabetic polyneuropathy (DPN). While transcutaneous electrical nerve stimulation (TENS) has proven effective in regressing sensory and motor impairments, its efficacy in improving postural control remains insufficiently studied. **Purpose:** To evaluate and compare the efficacy of direct peroneal and tibial high-frequency low-amplitude (HFLA) TENS and low-frequency high-amplitude (LFHA) TENS in correcting DPN-related postural disability, among European female patients without a documented history of falls, motor deficits, or pronounced electromyographic impairments, using computerized static posturography and the tandem walk test. **Materials and methods:** In this single-center, three-arm, randomized controlled trial (registration number: ISRCTN47534508, 3 December 2024), we conducted a longitudinal prospective analysis of European women with DPN-related postural disability. All enrolled patients were non-fallers with no motor deficits and baseline compound muscle action potential (CMAP) amplitudes of the peroneal and tibial nerves of at least 1.5 mV. The intervention groups received HFLA TENS (*n* = 24) or LFHA TENS (*n* = 25), while the control group underwent sham TENS (*n* = 24). Primary endpoints were assessed via static posturography and the tandem walk test (TWT); secondary endpoints were evaluated using hypoesthesia and pain evaluation, the Modified Clinical Test of Sensory Interaction in Balance (mCTSIB), and electromyography. Assessments were performed before treatment, immediately post-treatment, and at the conclusion of a 2-month follow-up period. **Results:** Comparative analysis incorporating the Bonferroni adjustment demonstrated that LFHA TENS is significantly superior to HFLA TENS. Post-treatment, LFHA TENS induced a reduction in envelope area by 20.7% under the eyes-open (EO) condition (*p* < α_adj_; α_adj_ = 0.0028) and 32.9% under the eyes-closed (EC) condition (*p* < α_adj_; α_adj_ = 0.0028), alongside a 16.6% decrease in the Romberg uotient (RQ) (*p* < α_adj_; α_adj_ = 0.0056). Furthermore, LFHA TENS elicited a significant 39.0% reduction in velocity of CoP sway (VCS) under the EO condition (*p* < α_adj_; α_adj_ = 0.0042), and decreased total CoP sway excursion by an average of 35.8% (EO) (*p* < α_adj_; α_adj_ = 0.0042) and 43.8% (EC) (*p* < α_adj_; α_adj_ = 0.0042) compared to baseline. In contrast, no statistically significant changes in these parameters were observed after HFLA TENS. Ultimately, LFHA TENS outperformed HFLA TENS in improving postural stability by 7.04% under the EO condition (*p* < α_adj_; α_adj_ = 0.0042) and by 25.5% under the EC condition (*p* < α_adj_; α_adj_ = 0.0042) in both the tandem walk test (TWT) and the Modified Clinical Test of Sensory Interaction on Balance (mCTSIB). Notably, a statistically significant increase in the CMAP amplitude of the affected peroneal nerves by 22.2% was observed exclusively following LFHA TENS treatment (*p* < α_adj_; α_adj_ = 0.0056). **Conclusions:** The clinical efficacy of direct peroneal and tibial TENS compared to sham stimulation in reducing postural disability during both static and dynamic conditions was established in European female patients with moderate-to-severe DPN and unremarkable EMG impairments. Comparative analysis reveals a clear therapeutic superiority of LFHA TENS over HFLA TENS, as evidenced by significantly greater improvements in both posturographic parameters (envelope area, total CoP excursion under EO and EC conditions, and VCS under the EO condition) and functional clinical tests (TWT and mCTSIB), demonstrating long-term stability for up to 2 months post-intervention.

## 1. Introduction

According to World Health Organization (WHO) data, the global prevalence of diabetes reached 14% among adults by 2022 [1], with over 50% affected by distal polyneuropathy (DPN) [2,3]. Alongside sensory and motor deficits, impaired postural control is a hallmark manifestation of this disease [4]. Evidence suggests that patients with DPN exhibit up to a 20-fold increase in fall risk compared to age-matched non-diabetic controls [5], demonstrating a strong correlation with diminished sensorimotor function in the lower extremities [6].

Postural control is defined as the feedback-driven, antigravity regulation of body orientation to maintain equilibrium [7] or as the process of positioning the body’s center of gravity (COG) within the center of pressure (CoP) [8]. This is achieved through complex, involuntary anticipatory and compensatory motor activity that aligns the body’s center of mass (COM) with the vector of gravity in response to the multimodal sensory integration of vestibular, visual, and somatosensory afferentation [9,10]. The primary outcomes of postural control are postural steadiness in static conditions and postural stability during dynamic movement [11]. Under normal conditions, the oscillations of body segments during postural maintenance do not exceed 2°, and the oscillations of the (CoP) remain under 2 cm [12].

The vestibular system maintains postural control by integrating sensory feedback from three-dimensional head rotations while concurrently monitoring gravitational vectors and transient linear accelerations [13]. In parallel, optic flow provides dynamic information regarding body position, self-motion and spatial orientation with respect to the visual coordinate system [14]. Additionally, somatosensory signals from proprioceptors in muscles and joints supply the central nervous system with information concerning the extent of lower-extremity muscle stretch induced by external perturbations [15]. Given that each of these sensory afferents has inherent limitations, the integration of these three modalities constitutes a robust and sophisticated sensory input for multilevel postural control centers [7].

While postural stability is maintained through the recruitment of trunk, neck, and limb muscle groups in response to sensory afferent signals and shifts in the body’s center of mass, the muscles of the lower extremities play the predominant role [16]. This process primarily engages slow-twitch muscle fibers, which are highly fatigue-resistant. Due to specific elastic and biomechanical properties, postural maintenance is achieved with minimal metabolic cost [17]. The most effective motor response for maintaining postural control is executed through distinct muscle synergies, commonly categorized as ankle and hip strategies [18]. Numerous studies suggest that these coordinated ankle–hip patterns can be modeled as a simplified hybrid control system, consisting of an active ankle component governed by sensory feedback and a passive hip component related to stiffness control [19]. Although the pure ankle strategy is more robust and energy-efficient due to a larger range of intermittent control gains, the hip strategy typically emerges when the ankle strategy becomes unstable. However, the stability of human erect posture is controlled in the sagittal plane by the ankle strategy, while in the frontal plane, control mainly relies on the hip strategy [20].

Postural control impairment is prevalent in patients with DPN, primarily driven by a combination of disrupted proprioceptive input and neurogenic denervation of the muscles of the lower legs and feet [21,22]. In healthy individuals, postural oscillations at the ankle joint during anterior–posterior sway typically occur within the 0.04–0.5 Hz range, whereas higher frequencies 0.5–2.5 Hz are more prominently manifested across individual anthropometric profiles, presenting a velocity of CoP sway of 9.2 ± 1.6 mm/s [8,23,24,25]. However, in patients with DPN, the mean velocity of CoP sway increases significantly. This acceleration is often accompanied by elevated excursion of CoP sway, predominantly in the anterior–posterior direction [26,27,28].

Electromyography (EMG) remains the gold standard for the precise and early diagnosis of DPN [29,30]. This method can assess not only the severity of DPN but also differentiate between the underlying axonal and demyelinating changes [26,27,28,29,30,31]. However, ENMG has notable limitations in evaluating the impairment of proprioceptive afferent pathways; consequently, this technique is not typically employed for the direct assessment of postural instability [26].

Among the simplest and most informative diagnostic methods for balance deficits in clinical practice is computerized posturography. It assesses the magnitude of CoP oscillations by determining the envelope area, velocity of CoP sway (VCS), total CoP sway excursion, and Romberg quotient. This information precisely captures postural control deviations from normative values and facilitates longitudinal monitoring of treatment efficacy [32]. Wang et al. demonstrated that the CoP can provide sufficient information on postural stability without EMG data, as CoP signals and low-frequency EMG activity are highly correlated [16].

Transcutaneous electrical nerve stimulation (TENS) has been shown in several studies to be effective in treating DPN, specifically by alleviating neuropathic pain, improving sensory perception, and restoring motor function [33,34,35,36,37,38,39]. The efficacy of this therapeutic modality is attributed to local, segmental, and central mechanisms [39]. Consequently, anatomical, neurophysiological, and functional improvements have been observed not only in sensory and motor nerve fibers but also within the central nervous system and the innervated muscles [38,39,40].

Previous studies comparing high-frequency and low-frequency TENS in the treatment of peripheral nervous system pathologies have demonstrated significant functional and neurophysiological recovery following low-frequency high-amplitude TENS (LFHA TENS) [41,42,43] alongside a pronounced analgesic effect after high-frequency low-amplitude TENS (HFLA TENS). However, a comparative analysis of these two modalities in the treatment of DPN-related postural disorders has not yet been conducted. To address this knowledge gap, the present study aimed to evaluate and compare the efficacy of direct peroneal and tibial HFLA versus LFHA TENS in correcting DPN-related postural disability among European female patients without a documented history of falls, motor deficits, or pronounced electromyographic impairments, using computerized static posturography and the tandem walk test.

## 2. Materials and Methods

### 2.1. Study Design and Participants

In this randomized, controlled, single-center, three-arm study, we conducted a longitudinal prospective analysis of patients with DPN-related postural disorders (Supplementary Materials). The intervention groups received HFLA TENS and LFHA TENS, while the control group underwent sham TENS. This study was carried out between 2024 and 2026 at the Department of Physiotherapy of RUDN University (Peoples’ Friendship University of Russia). The evaluations were conducted pre-treatment, post-treatment, and at a 2-month follow-up.

To minimize statistical variance, enhance intragroup homogeneity, and ensure data reliability, the study cohort was restricted to non-faller female participants matching the most prevalent anthropometric profile among women in Russia. Participants with a severe reduction in baseline CMAP amplitudes of peroneal and tibial nerves were explicitly excluded. This rigorous selection protocol was based on four methodological and biomechanical considerations:

- Sex-Related Posturographic Variations: Prior research demonstrates significant differences in normative values and deviation degrees in posturographic parameters between men and women [9,44,45].
- Biomechanical Constraints: According to the inverted pendulum model of human postural control, the frequency and amplitude of center of mass (COM) oscillations depend strictly on height, foot length, and body mass [46,47,48,49].
- Fall History: Patients with a history of frequent falls comprise 17% of the total diabetic peripheral neuropathy (DPN) population [50]. These individuals represent an advanced, severe course of DPN often confounded by diabetic comorbidities such as myopathies and proximal amyotrophy [6].
- Irreversibility of Axonal Damage: Severe baseline reductions in CMAP amplitude indicate an irreversible stage where electrophysiological recovery or meaningful improvement rarely occurs [33].

#### 2.1.1. Grouping Methodology

Eligibility Screening: A total of 141 patients with DPN-related postural disorders underwent initial eligibility screening. Of these, 59 patients were excluded: 48 did not meet the inclusion criteria and 10 patients declined to participate.

**Figure 1:**
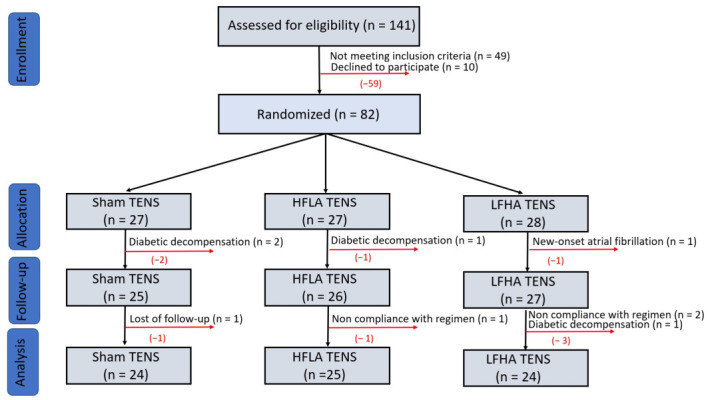
The CONSORT flow diagram. Note: TENS—transcutaneous electrical nerve stimulation; HFLA—high-frequency low-amplitude; LFHA—low-frequency high-amplitude.

Group Allocation: A total of 82 patients were randomized into three comparable groups: 27 in the control group, 27 in the HFLA TENS group, and 28 in the LFHA TENS group (Figure 1). During the initial stage, two patients from the control group were excluded due to diabetic decompensation. In the HFLA TENS group, one patient was excluded due to diabetic decompensation, while one patient from the LFHA TENS group was withdrawn due to new-onset atrial fibrillation.

During the follow-up period, an additional five patients were excluded. In the control group, one patient was lost to follow-up, while in the HFLA TENS group, another was excluded for non-compliance with the regimen. In the LFHA TENS group, three patients were withdrawn due to non-compliance (*n* = 2) and diabetic decompensation (*n* = 1). Consequently, the final analysis included 24 patients in the control group, 25 in the HFLA TENS group, and 24 in the LFHA TENS group.

#### 2.1.2. Participant Selection Criteria

Inclusion criteria:

1. Europeans.
2. Women aged 25 to 60 years.
3. Confirmed type 2 diabetes mellitus for more than 5 years.
4. Clinical manifestations of DPN for at least 3 years.
5. DPN diagnosis confirmed by ENMG.
6. DPN-related postural instability with no history of falls in the preceding 12 months.
7. No motor deficit in ankle dorsiflexion and plantarflexion.
8. Berg Balance Scale (BBS) scores < 49 points.
9. High postural instability, defined as an envelope area >150 mm and a Romberg quotient over 2.5.
10. Compound muscle action potential (CMAP) amplitudes of the peroneal and tibial nerves exceeding 1.5 mV.
11. Glycated hemoglobin (HbA1c) level < 7%.
12. Stature between 150 and 170 cm, foot length ranging from 230 to 270 mm, and a body mass index (BMI) within the 20–30 kg/m^2^ range.

Exclusion criteria:

1. History of epilepsy or seizure disorders.
2. Psychotic or major mental health disorders.
3. Clinically significant cardiac arrhythmias.
4. DPN of non-diabetic etiology.
5. Postural instability of other etiologies (e.g., vestibular or central nervous system disorders).
6. Current pregnancy.
7. Decompensation of hypertension and diabetes mellitus.
8. History of stroke or multiple sclerosis.

#### 2.1.3. Consent to Participate

All participants received a detailed explanation of the diagnostic and therapeutic procedures and provided written informed consent for both the medical interventions and their inclusion in the study. Prior to publication, participants reviewed the final manuscript and granted specific approval for the use of their data and study findings. The study was conducted on a voluntary basis; no financial compensation was provided to either the participants or the researchers.

#### 2.1.4. Ethical Consideration

The study protocol was approved by the Ethics Committee of the Russian Medical Dental Institute (Protocol No. 3111, dated 5 April 2022). This study was registered on 2 December 2024 (registration number: ISRCTN47534508). The first participant was enrolled on 4 December 2024. This research was conducted as part of the scientific program of the Department of Restorative Medicine and Neurorehabilitation at the Russian Medical Dental Institute and the Department of Physiotherapy at the Peoples’ Friendship University of Russia. All procedures were performed in accordance with the ethical standards of the Declaration of Helsinki (1964) and its subsequent amendments [51].

### 2.2. Sample Size Calculation

Based on the previous study by Puzin M. [52], the mean envelope area following TENS treatment for postural disorders in patients with distal polyneuropathy decreased significantly from 320.4 ± 85 mm^2^ to 235 ± 78 mm^2^ (*p* < 0.01), while no changes were observed in the sham TENS group. Using the ClinCalc.com calculator, a sample size of 21 patients was determined to achieve 90% statistical power, a Type I error rate of 0.01, and a significance level of 0.05.

### 2.3. Clinical Examination and Outcome Evaluation

All patients were under the supervision of a neurologist and an endocrinologist. All patients suffered from type 2 diabetes mellitus with a glycated hemoglobin level of less than 7%. All patients had suffered from DPN for at least 3 years, complicated by DPN-related postural disorders. The DPN diagnosis was confirmed by EMG, with no history of falls in the preceding 12 months. Patients were assessed at baseline, post-treatment, and at the end of the 2-month follow-up period.

#### 2.3.1. Primary Endpoints

##### Posturography

Computerized static posturography has been an established tool in postural diagnostics since the early 1970s [53]. This method has proven effective in diagnosing various conditions characterized by postural instability, particularly in patients with distal polyneuropathy of the lower extremities across different etiologies [30,54,55]. In the present study, postural control was assessed by measuring the following parameters under both eyes-open (EO) and eyes-closed (EC) conditions:

##### Envelope Area

Envelope area is defined as the 95% confidence ellipse area (measured in mm^2^) that encompasses approximately 95% of the CoP data points recorded during the trial. This parameter is calculated based on the bivariate distribution of CoP displacements in the anterior–posterior and mediolateral directions. Reported values for the envelope area vary significantly in the literature. Era et al. reported 66.03 ± 29.07 mm^2^, while Aghapour found 111.07 ± 61.97 mm^2^ during 20 s trials. When the testing duration was increased to 30 s, the area in young subjects reached 96.0 ± 68 mm^2^ (EO) and 113 ± 68 mm^2^ (EC) [25]. Conversely, Cavalheiro et al. reported higher values of 223 ± 129 mm^2^ (EO) and 326 ± 210 mm^2^ (EC) [56]. In healthy elderly subjects, these values were 173 ± 93 mm^2^ (EO) and 305 ± 250 mm^2^ (EC) [57].

##### Mean Velocity of CoP Sway

Mean velocity of CoP sway (VCS) represents the total path length traveled by the center of pressure (CoP) per second to maintain postural stability. Reported mean velocities range from 9.1 to 28.8 mm/s under EO conditions. The removal of visual input (EC) increases sway velocity by 10.7% [58], 14.5% [25], and 47.9% [56] in young adults, with the increase reaching up to 77.7% in the elderly population [59]. The maximal anterior–posterior distance (APD) and mediolateral distance (MLD) in healthy individuals are reported to be 16.3 ± 3.64 mm and 10.6 ± 3.64 mm under EO conditions, and 18.7 ± 6.78 mm and 9.93 ± 3.65 mm under EC conditions, respectively [58]. According to other authors, these values typically range between 12–19 mm in the sagittal plane and 8.77–9.33 mm in the frontal plane, showing no significant changes in the absence of visual input [47,59]. Literature data on normative values for the total excursion of center of pressure (CoP) sway are limited. Nevertheless, analysis of current MVCS in healthy subjects shows that total CoP excursion varies between 309 mm [60] and 525 mm [61] during a 30 s open-eyes standing test.

All patients were examined before and after treatment, as well as at the end of the follow-up period. The STABILO-MBN computerized stabilometric system (MBN, Moscow, Russia) was employed for posturographic testing (Registration No. FSR 2010/08455, 27 July 2021).

##### Tandem Walk Test

The tandem walk test (TWT) is a widely utilized clinical tool for screening patients with DPN [62]. In this study, patients were required to walk in a straight line in their socks, performing 10 heel-to-toe steps with no distance between the heel and the toe. The test was conducted under two conditions: eyes-open and eyes-closed. Prior to the assessment, participants received standardized instructions and performed several practice steps to familiarize themselves with the task. The primary outcome measure was the total number of correct tandem steps. Additionally, for patients who successfully completed all 10 steps, the time to completion was recorded in seconds.

#### 2.3.2. Secondary Endpoints

##### Sensory Examination

Hypoesthesia

Tactile sensation was assessed using 10 g (5.07 size) Semmes–Weinstein monofilament. The severity of tactile hypoesthesia was graded on a 5-point scale based on the proximal level of sensory loss:

- 5 points: At the level of the knee.
- 4 points: At the level of the mid-calf.
- 3 points: At the level of the ankle.
- 2 points: At the level of the mid-foot.
- 1 point: At the level of the toes.

Vibratory sensation was assessed using a calibrated Rydel–Seiffer tuning fork (128/64 Hz). Measurements were taken at the base of the hallux. The vibration threshold was determined by the visual intersection of the scale on the fork’s dampers at the moment the patient ceased to perceive the stimulus. All patients exhibited scores below 5/8, indicating a significant reduction in vibration perception. Since all patients initially presented with scores below 5, the vibration perception threshold was evaluated on a functional scale of 0 to 5, where 5 represented the maximum observed sensitivity and 0 indicated a complete lack of sensation. In cases where post-treatment measurements reached or exceeded 5 on the Rydel–Seiffer scale, they were recorded as 5 points for consistency. For statistical analysis, results from both lower extremities were averaged to derive a single mean value per patient.

Pain syndrome

The severity of pain syndrome was self-assessed by patients using a Visual Analogue Scale (VAS). Patients were asked to evaluate their symptoms over the preceding 24 h. Scores ranged from 0 to 10 points, where 10 represented the most severe symptoms and 0 indicated the complete absence of pain. Assessments were conducted at three time points: pre-treatment, immediately after treatment, and at the final follow-up.

##### Berg Balance Scale

The Berg Balance Scale (BBS) was developed by Katherine Berg in 1989. The BBS is a validated 14-item clinical instrument designed to assess static and dynamic balance through various functional tasks. Each item is scored on a 5-point scale (0–4), resulting in a maximum total score of 56, with lower scores indicating a higher risk of falls and impaired postural control [63]. This method serves as a robust diagnostic tool for evaluating postural instability in patients with DPN, yielding a sensitivity of 76.9% and a specificity of 90%. Clinical data show a distinct margin between groups: recurrent fallers recorded mean scores of 49.1 ± 3.2, whereas non-fallers maintained higher scores of 54.5 ± 2.5 [64]. The scale was administered at baseline to exclude patients at a high risk of falling. Based on this, patients with scores of 49 or higher were excluded from this study. Assessments were performed post-treatment and during the follow-up period; however, due to the ceiling effect, the changes were non-significant.

##### The Modified Clinical Test of Sensory Interaction in Balance

The Modified Clinical Test of Sensory Interaction in Balance (mCTSIB) was invented by Shumway-Cook and Horak in 1986 [65]. The mCTSIB is a standardized clinical assessment designed to quantify postural control under varying sensory conditions. It evaluates the effectiveness with which a patient integrates visual, vestibular, and somatosensory inputs to maintain equilibrium. The protocol consists of four standing conditions, each timed for a maximum of 30 s: Firm surface with eyes open; Firm surface with eyes closed; Foam surface with eyes open; Foam surface with eyes closed. Scoring is determined by the cumulative time (in seconds) the patient maintains balance across all subtests. While a total score of 120 s represents the physiological norm, lower scores serve as clinical indicators of pathological postural instability [65,66].

##### Electroneuromyography

The peroneal and tibial nerves were examined by ENMG at baseline and at the conclusion of the follow-up period. We evaluated the nerve conduction velocity (NCV) and compound muscle action potential (CMAP) amplitudes of the motor fibers, alongside the NCV and sensory nerve action potential (SNAP) amplitudes of the peroneal nerves [67]. This study exclusively included patients with axonal changes in whom these parameters remained recordable. The EMG device “Neuroexpeditor” from MBN was used (registration number: FSR 2010/07889).

### 2.4. Treatment Protocols

Direct labile stimulation of the peroneal and tibial nerves was performed. The control group received sham stimulation, while the remaining two groups underwent active TENS, with LFHA TENS applied in one group and HFLA TENS in the other. Detailed current characteristics are presented in Table 1. TENS therapy consisted of 20 sessions conducted every other day.

Sham TENS:

Control group patients received a course of sham TENS using an ineffective electrical pulse. The electrodes were placed on the anterior surface of the knee joint, with the cathode placed proximally and the anode distally.

**Figure 2:**
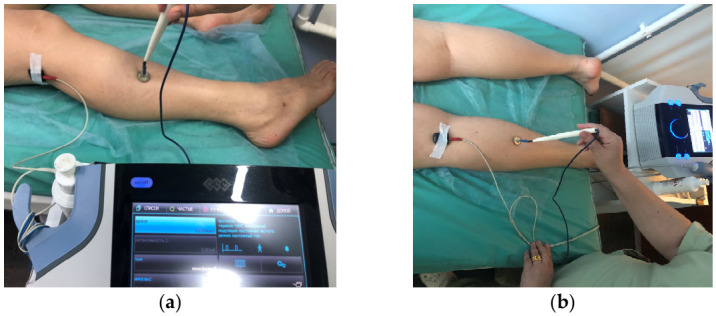
Technique of direct low-frequency high-amplitude transcutaneous electrical nerve stimulation of the peroneal (**a**) and tibial nerves (**b**). The cathode is fixed over the fibular tunnel when stimulating the peroneal nerve, and in the middle of the popliteal fossa when stimulating the tibial nerve. The anode (handheld pen-style electrode) is left unfixed and is moved from top to bottom in small increments, progressing from the proximal to the distal section.

LFHA TENS:
  - Peroneal Nerve Stimulation: With the patient in a supine position, the cathode was fixed over the fibular canal. The anode, a handheld pen-style electrode, was moved manually from the proximal to the distal segment in 10 cm increments. Stimulation at each point lasted 10 s. This procedure was repeated three times per nerve, with a mean total stimulation time of 5 min per nerve (Figure 2).
  - Tibial Nerve Stimulation: With the patient in a prone position, the cathode was fixed at the center of the popliteal fossa. The anode was a handheld pen-style electrode moved from the proximal to the distal segment in 10 cm increments. Stimulation at each point lasted 10 s. The procedure was repeated until the total stimulation time reached approximately 5 min per nerve (Figure 2).

HFLA TENS:
  - Peroneal Nerve Stimulation: the patient was placed in a supine position with the anode fixed over the fibular canal. A handheld pen-style cathode was moved manually in a proximal-to-distal direction in 10 cm increments. Stimulation at each site lasted 10 s and was repeated three times, resulting in a mean total stimulation time of 5 min per nerve.
  - Tibial Nerve Stimulation: The procedure was performed with the patient in the prone position with the anode fixed at the center of the popliteal fossa. A handheld pen-style cathode was moved manually in a proximal-to-distal direction in 10 cm increments. Each site was stimulated for 10 s, and the cycle was repeated until a total duration of approximately 5 min per nerve was achieved.

### 2.5. Statistical Analysis

In this randomized, controlled, single-blind, single-center, three-arm study, patients were allocated into three groups using SPSS for Windows (version 20) software. The patients were blinded to the treatment allocation, while the investigators were aware of the group assignments.

The primary endpoints in this research are the envelope area, MVCS, RQ and TWS. The secondary endpoints were hypoesthesia, pain syndrome, BBS, mCTSIB and CMAP amplitude of peroneal nerves. To ensure baseline comparability between the groups for the analysis of secondary endpoints, Propensity Score Matching was performed. To determine significant differences among the three groups prior to treatment, we used one-way ANOVA.

To assess significant differences, data were summarized as mean (M) and standard deviation (SD). Intragroup analysis was conducted to compare outcomes across three time points: pre-treatment, post-treatment, and follow-up. Additionally, an intergroup comparative analysis between the LFHA TENS and HFLA TENS groups was performed using Student’s *t*-test. To control for the family-wise Type I error rate under multiple comparisons, a Bonferroni post hoc correction was applied. Consequently, the *p*-values were adjusted, and the significance thresholds (α_adj_) were established as follows:

- For posturographic parameters and mCTSIB: α_adj_ = 0.0028 for intergroup comparisons (k = 18) and α_adj_ = 0.0042 for intragroup comparisons (k = 12).
- For the Romberg quotient: α_adj_ = 0.0056 for intergroup comparisons (k = 9) and α_adj_ = 0.0083 for intragroup comparisons (k = 6).
- For hypoesthesia, vibratory sensation, pain syndrome, and CMAP amplitude: α_adj_ = 0.0056 for both intergroup (k = 9) and intragroup (k = 9) comparisons.

Statistically significant results after the correction are indicated by an asterisk (*).

Furthermore, effect sizes were calculated using Cohen’s d to quantify the magnitude of the observed differences. Data normality was assessed using the Shapiro–Wilk test. The equality of variances of two groups was determined using the Levene test.

## 3. Results

### 3.1. Patient Demographics and Characteristics

This study enrolled female patients aged 47.9 ± 7.49 years with compensated type 2 diabetes and a mean DPN symptom duration of 9.63 ± 3.73 years (Table 2). To ensure posturographic reliability, strict anthropometric criteria were applied: stature of 160–175 cm (mean: 165.2 ± 4.30 cm), foot length of 225–255 mm (mean: 236.9 ± 6.79 mm), and a BMI of 20–30 kg/m^2^ (mean: 25.6 ± 2.94 kg/m^2^). The anthropometric profile of the women included in our study was characterized by an average stature, a normosthenic to slightly hypersthenic constitution, and a proportional foot length. This body constitution is highly prevalent among the female population in Russia.

All patients underwent neurological and endocrinological examinations. Each patient had well-controlled type 2 diabetes mellitus, maintaining a glycated hemoglobin (HbA1c) level below 7% [68]. Thus, the risk associated with other causes of postural dysfunction that typically develop secondary to uncontrolled hyperglycemia—such as cardiovascular dysfunction [69], diabetic encephalopathy [70], peripheral vestibular organ dysfunction [71,72], and diabetic retinopathy [73]—was excluded in all enrolled patients. The severity of diabetic peripheral neuropathy (DPN) was assessed by a neurologist. According to the Neuropathy Disability Score (NDS), all patients scored 6 points or higher, corresponding to moderate (6–8 points) and severe (9–10 points) stages. Specifically, the severe form was observed in 66.7% (*n* = 16) of the control group, 58.3% (*n* = 14) of the HFLA TENS group, and 68.0% (*n* = 17) of the LFHA TENS group, showing no statistically significant difference among the groups (χ^2^= 0.52, df = 2, *p* = 0.77).

All subjects exhibited pathological posturographic patterns. Prior to the intervention, the baseline eyes-open envelope area ranged from 120 to 220 mm^2^ (mean: 157.3 ± 24.4 mm^2^), while the eyes-closed envelope area ranged from 440 to 480 mm^2^ (mean: 463.1 ± 71.8 mm^2^). The RQ was registered between 2.5 and 4.0 (mean: 2.91 ± 0.42). In all patients, VCS ranged between 13.5 and 18.1 mm/s for the EO condition (mean: 15.7 ± 7.62 mm/s) and between 39.2 and 52.1 mm/s for the EC condition (mean: 45.9 ± 25.5 mm/s). Clinical assessment of postural impairment using the tandem walk test (TWT) revealed that the number of steps completed in the EO condition exceeded six steps, averaging 7.20 ± 1.00 steps. In contrast, the steps completed in the EC condition ranged from two to five, with a mean of 3.20 ± 0.78 steps. These data demonstrate marked postural instability resulting from proprioceptive impairment and subsequent compensatory visual dependence, which frequently develops in patients with DPN.

### 3.2. Primary Clinical Outcomes

#### 3.2.1. Dynamics of Posturographic Parameters

##### Envelope Area

All enrolled patients were diagnosed with severe DPN-related postural disorders. Baseline posturography showed a mean envelope area of 157.3 ± 24.4 mm^2^ in the eyes-open condition, with no significant intergroup differences. Following eye closure, resulted in a 2.91 ± 0.42-fold mean increase (Table 3).

LFHA-TENS application induced a significant reduction in the envelope area by 41% and 54.2% under both EO and EC conditions, accompanied by a decrease in the Romberg quotient to 2.2. Similarly, HFLA-TENS yielded comparable positive outcomes, reducing the envelope area by 21.0% (EO) and 30.7% (EC), while significantly lowering the Romberg quotient to 2.6.

During the 2-month follow-up period, the LFHA-TENS group demonstrated a sustained, progressive decline in the envelope area by 7.36% (EO) and 15.8% (EC); however, statistical significance was reached only under the EC condition. Concurrently, the Romberg quotient in this group continued to decrease, reaching 2.0. Conversely, the HFLA-TENS group exhibited a 10.3% (EO) and 14.6% (EC) increase in the envelope area, with the Romberg quotient rising to 2.7, thereby indicating negative dynamics during the 2-month follow-up. However, these differences proved non-significant.

A comparative analysis between the two modalities demonstrated the superiority of LFHA-TENS, which achieved greater envelope area reductions post-treatment (20.7% EO and 32.9% EC) and at 2-month follow-up (33.4% EO and 50.7% EC).

**Figure 3:**
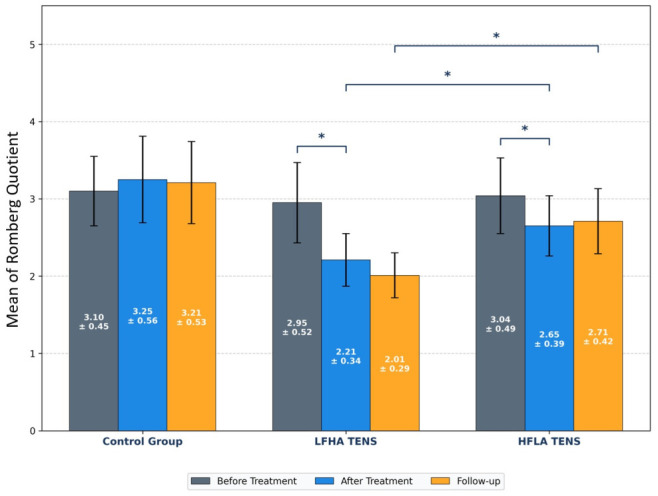
Dynamics of Romberg quotient in studied groups. Note: TENS—transcutaneous electrical nerve stimulation; LFHA—low-frequency high-amplitude; HFLA—high-frequency low-amplitude. After application of the Bonferroni correction, the adjusted significance threshold was set at α_adj_ = 0.0056 for intergroup comparisons and α_adj_ = 0.0083 for intragroup dynamics. The asterisk (*) denotes statistically significant differences (*p* < α_adj_).

Since the mean of individual row divisions of the envelope area of EC to EO mathematically differs from the ratio of their sample means, the average Romberg quotient (EC/EO) is illustrated separately in Figure 3. Comparative analysis demonstrated that the reduction in the RQ following LFHA TENS exceeded that of HFLA TENS by 16.6% post-treatment (t = 4.20, *p* < 0.001; α_adj =_ 0.0056, Cohen’s d = 1.20) and by 25.8% during 2-month follow-up (t = 6.81, *p* < 0.001; α_adj_ = 0.0056, Cohen’s d = 1.95).

In patients undergoing sham stimulation, the envelope area and Romberg quotient values showed no substantial changes either post-treatment or during the follow-up period.

##### Velocity of CoP Sway

Baseline values for mean VCS were elevated across all cohorts, reflecting postural instability. Pretreatment values were comparable between groups in both EO and EC conditions. Post-treatment, LFHA TENS demonstrated a significant reduction in the mean VCS by 39.0% (EO). However, the 32.5% reduction (EC) did not reach statistical significance. Although HFLA TENS also reduced mean VCS by 17.1% (EO) and 12.4% (EC), these changes lacked statistical significance due to the small sample size. During the 3-month follow-up, the reduction in mean VCS under EO and EC conditions remained stable across both TENS groups (Table 4).

##### Total CoP Sway Excursion

**Figure 4:**
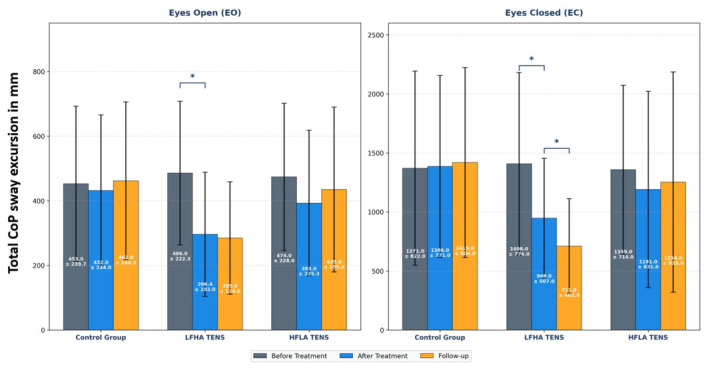
Dynamics of total CoP sway excursion in studied groups. Note: TENS—transcutaneous electrical nerve stimulation; LFHA—low-frequency high-amplitude; HFLA—high-frequency low-amplitude. After application of the Bonferroni correction, the adjusted significance threshold was set at α_adj_ = 0.0028 for intergroup comparisons and α_adj_ = 0.0042 for intragroup dynamics. The asterisk (*) denotes statistically significant differences (*p* < α_adj_).

At baseline, the mean total CoP sway excursion was 471.30 ± 227.1 for EO and 1378.50 ± 760.8 mm for EC, with no significant differences detected between the groups (Figure 4). These findings align with the results obtained for envelope area and mean VCS. Specifically, posturography revealed that maximum sagittal (anteroposterior) sway displacement exceeded frontal (mediolateral) displacement by 2.4-fold (EO) and 3.2-fold (EC). Similarly, total CoP path length in the anteroposterior direction was 9-fold (EO) and 12-fold (EC) greater than in the mediolateral direction. This pattern indicates postural instability resulting from proprioceptive deficits and impaired ankle flexor/extensor motor function. These characteristic impairments in patients with DPN are accompanied by an increased recruitment of the ankle strategy to maintain balance.

Post-treatment, LFHA TENS induced a decrease in total CoP excursion by 39% (t = 3.27, *p* = 0.001; α_adj_ = 0.0042, Cohen’s d = 0.91) for EO and 32.5% (t = 2.45, *p* = 0.01; α_adj_ = 0.0042, Cohen’s d = 0.70) for EC. Following HFLA TENS, total excursion decreased by 17.0% (EO) and 12.4% (EC); however, these changes were statistically insignificant. At follow-up, an additional 25% decrease was detected in the LFHA TENS group under the eyes-closed condition (t = 1.81, *p* = 0.04; α_adj_ = 0.0042, Cohen’s d = 0.52). Still, the change was moderate in nature and did not reach statistical significance.

Thus, a reduction in the total CoP sway excursion is observed exclusively following LFHA TENS post-treatment, with the therapeutic effect remaining stable and sustained during the 2-month follow-up.

#### 3.2.2. Dynamics of Tandem Walk Test

Moderate difficulties were observed during the TWT under the eyes-open condition, with patients completing an average of 7.19 ± 1.00 steps (Table 5). In contrast, the eyes-closed condition caused substantial impairment in the majority of participants, markedly decreasing the step count to 3.20 ± 0.78 steps. At baseline, no significant intergroup differences were detected under either the eyes-open (F = 0.09, *p* = 0.914) or eyes-closed (F = 0.07, *p* = 0.934) conditions.

Both TENS modalities led to an increase in the number of steps completed during the TWT. Specifically, the step count rose by 33.7% (EO) and 71.9% (EC) after LFHA TENS, compared to 22.2% (EO) and 30.4% (EC) following HFLA TENS. Subsequently, no deterioration was observed during the follow-up period in any of the studied groups.

Overall, LFHA TENS was associated with greater improvements in gait stability compared to HFLA TENS, showing an advantage of 10.9% (EO) and 33.5% (EC) immediately post-treatment, and 10.9% (EO) and 34.0% (EC) at the end of the 2-month follow-up.

Following sham stimulation, no significant changes were observed either post-treatment or at the end of the follow-up period.

### 3.3. Secondary Clinical Outcomes

#### 3.3.1. Dynamics of Hypoesthesia and Pain Syndrome

Hypoesthesia

**Figure 5:**
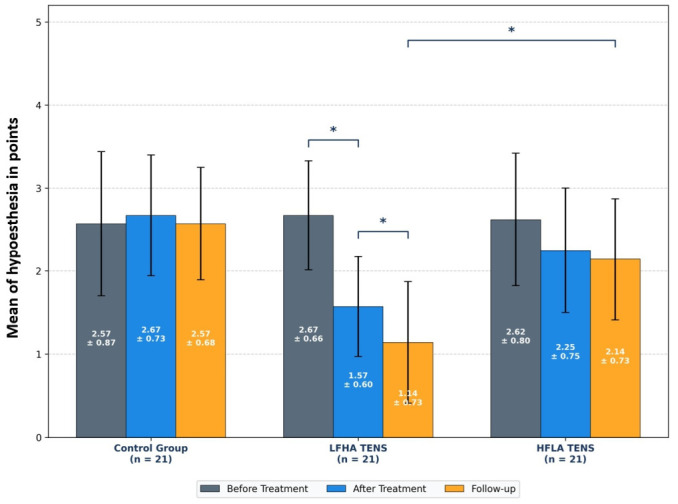
Dynamics of hypoesthesia in studied groups. Note: TENS—transcutaneous electrical nerve stimulation; LFHA—low-frequency high-amplitude; HFLA—high-frequency low-amplitude. After application of the Bonferroni correction, the adjusted significance threshold was set at α_adj_ = 0.0056 for intergroup comparisons and α_adj_ = 0.0056 for intragroup dynamics. The asterisk (*) denotes statistically significant differences (*p* < α_adj_).

The baseline hypoesthesia across all groups was 2.62 ± 0.77 points, with no statistically significant differences between the groups (F = 0.078, *p* = 0.925). This baseline average indicates that for the majority of patients, the level of hypoesthesia was, on average, located at the ankle joint level and below (Figure 5). Following the effective TENS therapy, the level of tactile hypoesthesia significantly decreased by 41.2% in the LFHA TENS group (t = 6.45, *p* < 0.001; α_adj_ = 0.0056, Cohen’s d = 1.41), and significantly did not change in the HFLA TENS group (t = 2.12, *p* = 0.046; α_adj_ = 0.0056, Cohen’s d = 0.46). During the follow-up period, these positive results continued to improve in the LFHA TENS group, showing an additional 27.3% reduction compared to post-treatment values (t = 4.44, *p* = 0.0001; α_adj_ = 0.0056, Cohen’s d = 1.37). On the other hand, the hypoesthesia level in the HFLA TENS group remained stable without substantial dynamics (t = 0.73, *p* = 0.473; α_adj_ = 0.0056, Cohen’s d = 0.16). No statistically significant difference could be identified between the treatment outcomes of the two TENS groups immediately post-treatment (t = 1.97, *p* = 0.056, Cohen’s d = 0.61), despite the fact that the clinical results of LFHA TENS exceeded those of HFLA TENS by 30.2%. Nevertheless, when comparing the therapeutic outcomes between the groups during the follow-up period, LFHA TENS proved to be highly superior to HFLA TENS by 46.7% (t = 4.43, *p* < 0.0001; α_adj_ = 0.0056, Cohen’s, d = 1.37).

**Figure 6:**
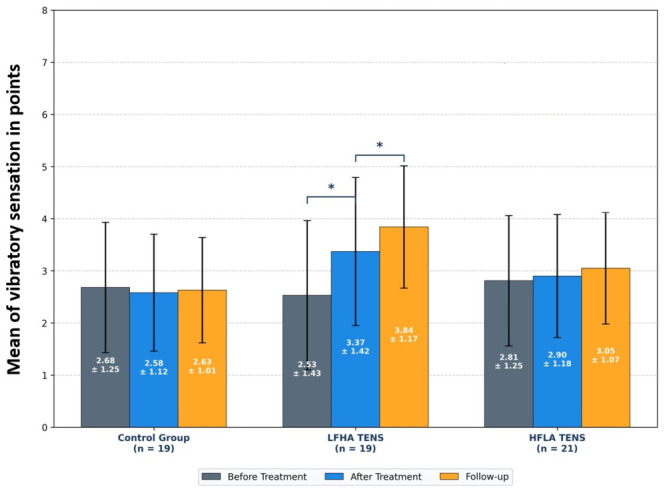
Dynamics of vibratory sensation in studied groups. Note: TENS—transcutaneous electrical nerve stimulation; LFHA—low-frequency high-amplitude; HFLA—high-frequency low-amplitude. After application of the Bonferroni correction, the adjusted significance threshold was set at α_adj_ = 0.0056 for intergroup comparisons and α_adj_ = 0.0056 for intragroup dynamics. The asterisk (*) denotes statistically significant differences (*p* < α_adj_).

Investigation of vibratory sensation using an eight-point tuning fork scale revealed a baseline mean of 2.62 ± 0.94 points across all cohorts (Figure 6), with no statistically significant differences between the groups (F = 0.057, *p* = 0.945). A statistically significant improvement in vibratory sensation was observed exclusively in the LFHA TENS group. Post-treatment, vibratory sensation significantly increased by 30.6% (t = −3.73, *p* = 0.002; α_adj_ = 0.0056, Cohen’s d = 0.86), with a continued increase of an additional 20.3% (t = 3.20, *p* = 0.005; α_adj_ = 0.0056, Cohen’s d = 0.73) during the 3-month follow-up.

No statistically significant alterations were detected following sham stimulation, remaining stable post-treatment and throughout the follow-up period.

Pain syndrome

**Figure 7:**
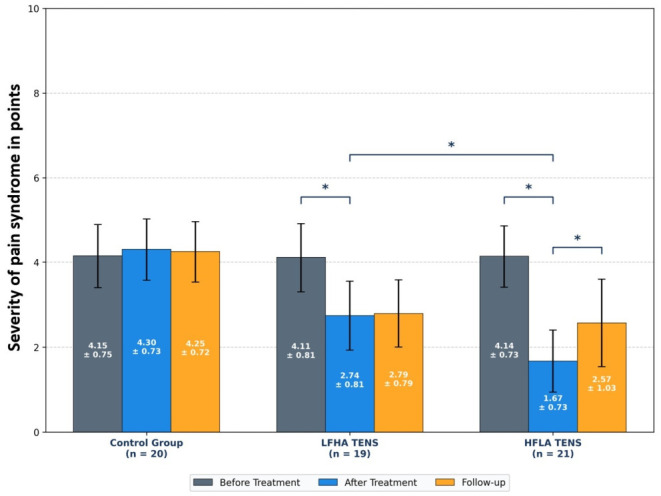
Dynamics of pain syndrome in studied groups. Note: TENS—transcutaneous electrical nerve stimulation; LFHA—low-frequency high-amplitude; HFLA—high-frequency low-amplitude. After application of the Bonferroni correction, the adjusted significance threshold was set at α_adj_ = 0.0056 for intergroup comparisons and α_adj_ = 0.0056 for intragroup dynamics. The asterisk (*) denotes statistically significant differences (*p* < α_adj_).

Across the investigated groups, the maximum value of pain syndrome did not exceed 5 points (Figure 7). The baseline mean for all studied patients was 4.13 ± 0.76 points, with no significant intergroup differences (F = 0.184, *p* = 0.832). Following LFHA TENS, the severity of the symptoms significantly decreased by 32.3% (t = 6.42, *p* < 0.001; α_adj_ = 0.0056, Cohen’s d = 1.47), and after HFLA TENS, by 60.1% (t = 11.45, *p* < 0.001; α_adj_ = 0.0056, Cohen’s d = 2.50). During 2 months after treatment, a statistically significant recurrence of symptoms was observed in the HFLA TENS group, with scores increasing by 53.9% from post-treatment levels (t = 4.85, *p* < 0.001; α_adj_ = 0.0056, Cohen’s d = −1.06). Meanwhile, in the LFHA TENS group, the therapeutic effect demonstrated a prolonged, stable, and positive analgetic effect at the end of the 2-month follow-up (t = 0.25, *p* = 0.805; α_adj_ = 0.0056, Cohen’s d = −0.06). Interestingly, a statistically significant advantage in analgetic effect was found immediately after HFLA TENS, with scores remaining 39.1% lower than those of the LFHA TENS group (t = 4.21, *p* < 0.0001; α_adj_ = 0.0056, Cohen’s d = 1.39). However, by the end of the 2-month follow-up, this achieved advantage diminished due to symptom recurrence. Consequently, the severity of pain syndrome in the HFLA TENS and LFHA TENS groups failed to show any meaningful variation (t = 0.74, *p* = 0.464; α_adj_ = 0.0056, Cohen’s d = 0.23).

Sham stimulation produced no significant changes post-treatment or during follow-up.

#### 3.3.2. The Modified Clinical Test of Sensory Interaction in Balance

**Figure 8:**
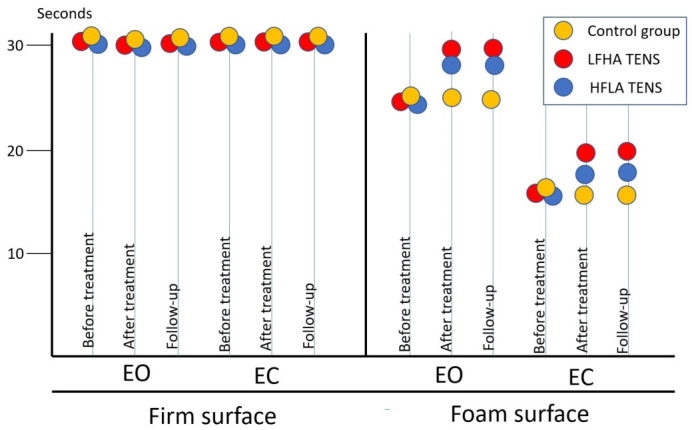
Dynamics of the modified clinical test of sensory interaction in balance on a foam surface across groups.

In mCTSIB, all patients achieved the maximum 30 s duration in the first two conditions (standing on a firm surface with eyes open and closed). In contrast, a different trend was observed on the foam surface (Table 6, Figure 8). Specifically, the standing duration on the foam surface with eyes open approached the upper normal limit, averaging 25.7 ± 1.74 s, but decreased to an average of 16.4 ± 1.92 s with eyes closed. Post-treatment, this duration increased by 12.5% (EO) and 30.5% (EC) after LFHA TENS and by 9.45% (EO) and 11.7% (EC) after HFLA TENS. At 3-month follow-up, standing time remains stable after LFHA TENS and HFLA TENS. Comparative analysis demonstrated that LFHA TENS outperformed HFLA TENS in maintaining postural stability in the eye-closed condition by 17.6% post-treatment and by 24.9% at the end of the follow-up period.

Patients in the sham stimulation group demonstrated no reliable changes immediately after treatment or at the end of follow-up.

#### 3.3.3. Dynamics of Electromyography Changes

In accordance with the inclusion criteria, all participants demonstrated signs of peroneal axonal neuropathy, presenting a decline in peroneal nerve CMAP amplitude between 1.5 mV and 3.0 mV. Importantly, the prior literature defines the lower limit of normal for peroneal CMAP amplitude as 3.0 mV [36,41]. Due to anatomical characteristics, tibial nerve lesions develop less frequently and are clinically less prominent than those of the peroneal nerve. Moreover, normal tibial CMAP amplitudes are often 2–3 times higher than the lower limit of normal of CMAP amplitude for this nerve (3.5 mV). Therefore, evaluating a tibial amplitude slightly above 3.5 mV remains challenging, as it could indicate either a normal baseline value or a regression from a significantly higher individual level. For this reason, we focused exclusively on analyzing the dynamics of peroneal nerve CMAP amplitudes.

**Figure 9:**
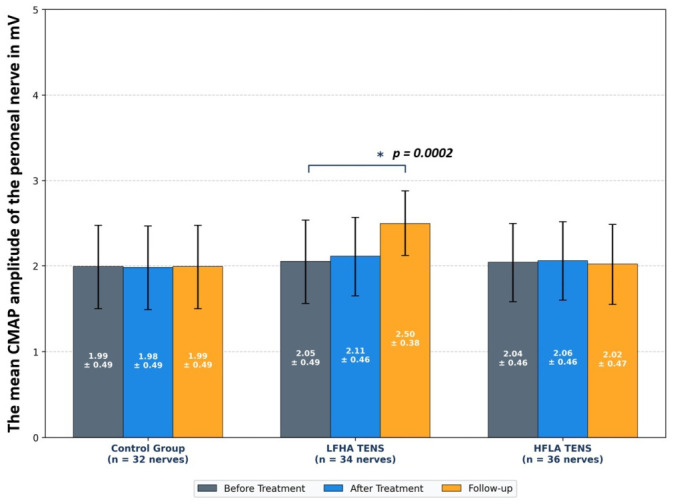
Dynamics of peroneal nerve compound muscle action potential amplitudes in studied groups. Note: CMAP—compound muscle action potential; TENS—transcutaneous electrical nerve stimulation; LFHA—low-frequency high-amplitude; HFLA—high-frequency low-amplitude. After application of the Bonferroni correction, the adjusted significance threshold was set at α_adj_ = 0.0056 for intergroup comparisons and α_adj_ = 0.0056 for intragroup dynamics. The asterisk (*) denotes statistically significant differences (*p* < α_adj_).

The results of our study demonstrated that the baseline mean of peroneal nerve CMAP amplitudes across all evaluated cohorts was 2.03 ± 0.47 mV, with no remarkable significant difference observed across the groups (F = 0.129, *p* = 0.879). Following the intervention, neither the control group nor the HFLA TENS group demonstrated any statistically detectable changes immediately post-treatment or during the long-term follow-up period (t = 0.35, *p* = 0.729, Cohen’s d = 0.06). In contrast, at the end of the follow-up period, a significant increase of 21.95% in peroneal nerve CMAP amplitude was registered after LFHA TENS compared to the baseline level (t = 4.23, *p* < 0.0002; α_adj_ = 0.0056, Cohen’s d = 1.03) (Figure 9).

### 3.4. Side Effects

No adverse effects were reported for either LFHA TENS or HFLA TENS.

## 4. Discussion

The role of lower-extremity neuropathies in the development of postural disability is extensively documented [6,10,21,22,27,74,75]. Likewise, multiple studies have examined the efficacy of TENS, including direct stimulation of the peroneal and tibial nerves, in reducing clinical and neurophysiological deficits secondary to DPN [2,38,39,40,41,42,43,44,45,46,76,77,78]. However, to our knowledge, no prior studies have evaluated the effectiveness of different modalities of direct TENS in managing DPN-related postural disorders.

In our study, a comparative analysis was performed among sham TENS, LFHA TENS, and HFLA TENS, which were applied 20 times every other day. To assess postural disability, computerized posturography and postural clinical tests were conducted. Additionally, we clinically evaluated the dynamics of pain syndrome and hypoesthesia, alongside EMG changes in peroneal nerve fibers. Postural and neurological examinations were performed before treatment, after treatment, and 2 months after TENS therapy was completed.

To eliminate inaccuracies in the obtained posturography results, specific clinical criteria were established based on sex, age, anthropometric characteristics, and ENMG changes. Consequently, the study cohort comprised European female participants presenting with moderate and severe DPN.

The results of our study demonstrated that both direct peroneal and tibial HFLA and LFHA TENS were highly effective in reducing DPN-related postural disorders compared to sham TENS. However, some differences were observed in the degree of postural control improvement and clinical outcomes of DPN, which may be directly linked to the physiological mechanisms of their respective therapeutic effects.

Efficiency of HFLA TENS

In our study, postural control improvement among patients with DPN-related postural disorders undergoing HFLA TENS was demonstrated by a significant reduction in envelope area by 21.0% (*p* < α_adj_; α_adj_ = 0.0042) under the EO condition and by 30.7% (*p* < α_adj_; α_adj_ = 0.0042) under the EC condition. Additionally, a significant average improvement in TWT and mCTSIB tests was observed, with values increasing by 15.8% (*p* < α_adj_; α_adj_ = 0.0042) under the EO condition and 21.0% (*p* < α_adj_; α_adj_ = 0.0042) under the EC condition. This effect was sustained without significant deterioration for 2 months after treatment completion.

The reduction in postural disorders was accompanied by a robust analgesic effect. We hypothesize that this factor underlies the improvement in postural stability observed following HFLA TENS treatment. Prior studies have demonstrated that HFLA TENS exerts a marked analgesic effect through the segmental activation of substantia gelatinosa cells in the spinal cord dorsal horns [2,79,80,81]. This pronounced segmental analgesic effect, when applied as a long-term repetitive intervention, progressively induces the suppression of central sensitization. However, the immediate segmental analgesic effect during the treatment session is transient. Consequently, after a 59.7% reduction at the end of treatment (*p* < α_adj_; α_adj_ = 0.0056), pain intensity increased by the end of the follow-up period, returning to a level 37.9% below the pre-treatment baseline (*p* < α_adj_; α_adj_ = 0.0056). Nevertheless, these short-term follow-up changes did not lead to a statistically significant negative trend in posturographic and clinical outcomes. According to previous reports, postural disability is more pronounced in patients with the painful form of DPN. Furthermore, numerous studies have hypothesized that sensory afferentation is ignored in chronic pain syndromes, a phenomenon known as “nondermatomal somatosensory deficits” [82,83,84,85,86,87]. This phenomenon, in turn, may contribute to postural instability.

Efficiency of LFHA TENS

Postural control improvement following LFHA TENS was highly pronounced, with the reduction in envelope area exceeding the outcomes of HFLA TENS by 2.0-fold under the EO condition (*p* < α_adj_; α_adj_ = 0.0028) and by 1.8-fold under the EC condition (*p* < α_adj_; α_adj_ = 0.0028). Furthermore, this positive effect extended to both the velocity of sway (VCS) under the EO condition (*p* < α_adj_; α_adj_ = 0.0042) and total CoP excursion metrics under both EO and EC conditions (*p* < α_adj_; α_adj_ = 0.0042), which manifested uniquely following LFHA TENS treatment. A similar superiority was observed across clinical tests, where LFHA TENS demonstrated an average improvement that was 1.44 times greater under the EO condition (*p* < α_adj_; α_adj_ = 0.0056) and 2.65 times greater under the EC condition (*p* < α_adj_; α_adj_ = 0.0056). This superiority of LFHA TENS in improving postural stability is mediated by several underlying mechanisms:

Analgesic efficacy

According to the gate control theory of pain, the immediate analgesic effect mediated by the stimulation of fast-conducting input (myelinated sensory afferents) directly correlates with the frequency of electrical stimulation. This phenomenon is clearly observed at the end of the treatment course and after each individual HFLA TENS session, highlighting the short-term advantages of HFLA TENS. However, LFHA TENS provides a prolonged and stable analgesic response, which does not significantly differ from that of HFLA TENS at the end of the 2-month follow-up period.

Numerous experimental and clinical studies have extensively investigated and discussed the analgesic efficacy of LFHA TENS. Accumulating evidence from these investigations establishes that the analgesic effect of LFHA TENS, in addition to the segmental effect [88,89], relies on central analgesic mechanisms [89,90]. The transmission of impulses to higher brain structures via spinal cord pathways, or parasegmentally, stimulates μ-opioid receptors on specific neurons in the ventrolateral periaqueductal gray and rostral ventromedial medulla, leading to a pronounced inhibition of nociceptive afferent signaling via the spinothalamic tract [2,91,92]. Moreover, LFHA TENS induces the release of potent endogenous analgesic substances, including endogenous opioids (endorphins and enkephalins) and serotonin, and activates descending pain inhibitory pathways [2,93,94]. A recent publication demonstrated the activation of the primary sensorimotor cortex after TENS and its relationship with the medial prefrontal cortex, both of which modulate the descending pain inhibitory system. These central mechanisms elicit a distinct, spatially diffuse analgesic effect [89,94]. Furthermore, the local recovery and regenerative effect within the neurostimulation zone leads to a significant reduction in ectopic pacemaker activity and the suppression of peripheral sensitization mechanisms [43,95,96,97,98].

Recovery and regenerative effect

In reducing hypoesthesia, LFHA TENS was shown to be highly effective, resulting in a 57.3% (*p* < α_adj_; α_adj_ = 0.0056) alleviation of tactile sensory deficit and a 51.8% improvement in vibratory sensation (*p* < α_adj_; α_adj_ = 0.0056) by the end of the second month post-treatment compared to pre-treatment levels. Notably, the improvement in vibratory sensation is closely associated with the function of proprioceptive afferents. Concurrently, tactile and vibratory sensation recovery was completely absent in the HFLA TENS group. These improvements were not merely functional but also structural in nature, as evidenced by a significant increase in the compound muscle action potential (CMAP) of the peroneal nerves.

The recovery and regenerative effect of LFHA TENS was demonstrated in our previous studies [41,42,43,99] and reported in other publications [100,101]. Several authors have demonstrated that LFHA TENS promotes a cascade of regenerative mechanisms in affected nerves and increases their resistance to ischemia and injury. This occurs by improving microcirculation and angiogenesis [41,102] within the electrical pulse application zones, stimulating the accumulation of growth factors and brain-derived neurotrophic factor [103], and accelerating the reinnervation process [42,104,105]. The results of our study successfully demonstrated the efficacy of LFHA TENS on proprioception, as evidenced by the positive dynamics observed in vibratory sensation. This improvement enhances the intensity and velocity of coordinated non-nociceptive afferent signaling, thereby providing more precise and rapid center of pressure (CoP) coordinates.

Furthermore, LFHA TENS induced a moderate 22.2% (*p* < α_adj_; α_adj_ = 0.0056) increase in the peroneal nerve CMAP amplitude by the end of the second month of follow-up. This neurophysiological enhancement likely reflects an accelerated reinnervation process alongside an increase in the quantity of active motor units. Concurrently, these findings suggest a reduction in the number of fatigued muscle fibers resulting from the neuromuscular disorder. Collectively, these structural and functional adaptations mitigate protective movement strategies and optimize muscle tone during both standing and walking.

Another potential mechanism underlying the therapeutic effect of LFHA TENS is the enhancement of fine motor control, which has been documented in prior research addressing neuropathy complications with preserved motor function [43]. This effect is associated with the activation of coordinated muscle control and an improvement in neuromuscular control at both peripheral and central levels [106].

Recent studies have established that patients with DPN develop functional and structural changes in the primary sensorimotor cortex and somatosensory pathway, which correlate with the severity of peripheral neuropathy [95,107]. Interestingly, researchers have identified a reduced gray matter volume in the deep gray matter nuclei and dorsolateral prefrontal cortex [95], as well as thinning of the cingulate, insula, and prefrontal cerebral cortex [96,97]. These changes become an additional cause of postural impairments in patients with DPN [98]. A body of literature has demonstrated the high efficacy of TENS in restoring motor cortex excitability, leading to improved postural control and interlimb coordination [108,109].

## 5. Limitations

Several limitations of this study should be noted. First, the cohort was exclusively restricted to European female participants due to documented sex-related differences in normative posturographic parameters. Consequently, our findings cannot be directly generalized to male patients, and further research is required to evaluate the efficacy of these interventions in the male population. Furthermore, the study included only women matching the most prevalent regional anthropometric profile in Russia. Specifically, individuals taller than 170 cm, with a foot length exceeding 270 mm, or a body mass index (BMI) over 30 kg/m^2^ were excluded. While these specific anthropometric variations are unlikely to alter the underlying therapeutic efficacy of the treatments, they could independently influence baseline posturographic metrics, limiting the baseline applicability of our results to these subgroups. Additionally, this investigation excluded patients with severe EMG impairments, specifically those with a baseline CMAP amplitude below 1.5 mV. Therefore, the demonstrated therapeutic benefits do not apply to individuals with advanced axonal damage. Additional studies are warranted to systematically investigate these treatment methods in patients with severe EMG disorders. A major limitation of this study is the relatively short follow-up period (2 months). Future studies with longer follow-up are needed to assess the long-term sustainability of the treatment effects. Finally, a limitation of our study is that only a single-blind protocol could be implemented. Blinding the investigators was not feasible due to the nature of the intervention, as they had to manually perform the stimulation and select the parameters.

## 6. Conclusions

The clinical efficacy of direct peroneal and tibial TENS compared to sham stimulation in reducing postural disability during both static and dynamic conditions was established in European female patients with moderate-to-severe DPN and unremarkable EMG impairments. Comparative analysis reveals a clear therapeutic superiority of LFHA TENS over HFLA TENS, as evidenced by significantly greater improvements in both posturographic parameters (envelope area, total CoP excursion under EO and EC conditions, and VCS under the EO condition) and functional clinical tests (TWT and mCTSIB), demonstrating long-term stability for up to 2 months post-intervention.

While the post-treatment enhancement of postural stability following HFLA TENS is predominantly mediated by its robust but non-sustained analgesic effect, the superiority of LFHA TENS is associated with achieving a stable analgesic response, on the one hand, and a noticeable improvement in the ankle strategy, which is directly facilitated by peripheral sensory and motor recovery, on the other hand. This functional recovery is further corroborated by a significant neurophysiological increase in the CMAP amplitude of the affected peroneal nerves, which was not registered after HFLA TENS.

Further research is required to systematically evaluate the clinical efficacy of direct TENS in male cohorts, diverse ethnic groups, and patients with severe electromyographic abnormalities.

## Figures and Tables

**Table 1:** Electrical pulse characteristics of TENS treatment across the three study groups.

	Waveform	Frequency	Duration	Amplitude
Sham TENS	Monophasic square shape	1 Hz	50 ms	Sensory threshold
HFLA TENS		50 Hz	50 ms	3 mA above the sensory threshold
LFHA TENS		1 Hz	200 ms	3 mA above the motor threshold

**Table 2:** Characteristics of the participants.

Characteristic	Control Group	LFHA TENS	HFLA TENS	(One-Way ANOVA)	
				F *	*p* **
Number of participants, *n*	24	25	24		
Female	24	25	24		
Age (year)	47.4 ± 7.05	49.2 ± 7.15	46.9 ± 8.24	0.64	0.53
Foot length (mm)	235.0 ± 6.71	239.0 ± 7.15	236.7 ± 6.50	2.14	0.12
Stature (cm)	165.6 ± 3.51	164.8 ± 4.24	165.1 ± 5.01	0.22	0.80
BMI (kg/m^2^)	25.3 ± 2.89	25.1 ± 3.11	26.5 ± 2.68	1.64	0.20
Duration of DPN	8.83 ± 3.35	10.5 ± 4.10	9.51 ± 3.65	1.21	0.30
Envelope area EO (mm^2^)	150.4 ± 73.5	165.4 ± 77.1	155.8 ± 72.3	0.26	0.71
Envelope area EC (mm^2^)	458.8 ± 71.9	468.6± 76.8	461.7 ± 66.1	0.12	0.89
Velocity of CoP sway EO (mm/second)	15.1 ± 7.99	16.2 ± 7.41	15.8 ± 7.60	0.13	0.87
Velocity of CoP sway EC (mm/second)	45.7 ± 27.4	46.8 ± 25.8	45.3 ± 23.8	0.02	0.98
Romberg quotient	3.02 ± 0.43	2.81 ± 0.41	2.91 ± 0.43	1.50	0.23
Tandem walk test EO (steps)	7.20 ± 1.06	7.25 ± 1.03	7.16 ± 0.96	0.05	0.95
Tandem walk test EC (steps)	3.25 ± 0.89	3.20 ± 0.58	3.16 ± 0.86	0.08	0.92

Note: TENS—transcutaneous electrical nerve stimulation; LFHA—low-frequency high-amplitude; HFLA—high-frequency low-amplitude; DPN—diabetic peripheral neuropathy; CoP—centre of pressure. Mean ± standard deviation (SD), * F—statistic, ** *p*—*p*-value (one-way ANOVA).

**Table 3:** Dynamics of posturographic envelope area across groups.

	Before Treatment		After Treatment		Follow-Up			Significant Difference		
	EO^1^	EC^1^	EO^2^	EC^2^	EO^3^	EC^3^	EO^2^:EO^1^	EO^3^:EO^2^	EC^2^:EC^1^	EC^3^:EC^2^
Control group (*n* = 24)	150.4 ± 23.5 95% CI [140.5—160.3]	458.8 ± 71.9 95% CI [428.4–489.2]	148.8 ± 29.1 95% CI [136.5–161.1]	477.6 ± 93.4 95% CI [438.2–517.0]	151.1 ± 24.1 95% CI [140.9–161.3]	480.1 ± 86.6 95% CI [443.5–516.7]	t = 0.22 *p* = 0.42 Cd = 0.06	t = 0.31 *p* = 0.38 Cd = 0.08	t = 0.78 *p* = 0.22 Cd = 0.22	t = 0.09 *p* = 0.46 Cd = 0.03
LFHA TENS group (*n* = 25)	165.4 ± 27.1 95% CI [154.2–176.6]	468.6 ± 76.8 95% CI [436.9–500.3]	97.6 ± 18.0 95% CI [90.2–105.0]	214.7 ± 39.6 95% CI [198.4–231.0]	90.4 ± 15.8 95% CI [83.9–96.9]	180.8 ± 31.6 95% CI [167.8–193.8]	t = 10.4 *p* < 0.0001 * Cd= 2.94	t = 1.50 *p* = 0.07 Cd = 0.43	t = 14.7 *p* < 0.0001 * Cd = 4.15	t = 3.40 *p* = 0.001 * Cd = 0.96
HFLA TENS group (*n* = 24)	155.8 ± 22.3 95% CI [146.4–165.2]	461.7 ± 66.1 95% CI [433.8–489.6]	123.1 ± 19.2 95% CI [115.0–131.2]	320.1 ± 49.8 95% CI [299.1–341.1]	135.8 ± 20.5 95% CI [127.1–144.5]	346.7 ± 52.3 95% CI [324.6–368.8]	t = 5.42 *p* < 0.0001 * Cd = 1.57	t = 2.18 *p* = 0.016 Cd = 0.64	t = 8.38 *p* < 0.0001 * Cd = 2.41	t = 1.80 *p* = 0.038 Cd = 0.52
	Significant differences between LFHA TENS and HFLA TENS groups									
	t = 1.35 *p* = 0.18 Cd = 0.39	t = 0.34 *p* = 0.74 Cd = 0.10	t = 4.82 *p* ≤ 0.0001 * Cd = 1.37	t = 8.22 *p* < 0.0001 * Cd = 2.35	t = 8.70 *p* < 0.0001 * Cd = 2.48	t = 13.5 *p* < 0.0001 * Cd = 3.86				

Note: EO^1^—eye open before treatment; EO^2^—eye open after treatment; EO^3^—eye open at the end of follow-up; EC^1^—eye closed before treatment; EC^2^—eye closed after treatment; EC^3^—eye closed at the end of follow-up; TENS—transcutaneous electrical nerve stimulation; LFHA—low-frequency high-amplitude; HFLA—high-frequency low-amplitude, t—test statistic, *p*—*p*-value, Cd—Cohen’s d; mean ± standard deviation (SD), confidence interval in mm^2^. After application of the Bonferroni correction, the adjusted significance threshold was set at α_adj_ = 0.0028 for intergroup comparisons and α_adj_ = 0.0042 for intragroup dynamics. The asterisk (*) denotes statistically significant differences (*p* < α_adj_).

**Table 4:** The dynamics of mean velocity of CoP sway across groups.

	Before Treatment		After Treatment		Follow-Up		Significant Difference			
	EO^1^	EC^1^	EO^2^	EC^2^	EO^3^	EC^3^	EO^2^:EO^1^	EO^3^:EO^2^	EC^2^:EC^1^	EC^3^:EC^2^
Control group (*n* = 24)	15.1 ± 7.99 95% CI [11.9–18.3]	45.7 ± 27.4 95% CI [34.7–56.7]	14.4 ± 7.80 95% CI [11.3–17.5]	46.2 ± 25.7 95% CI [35.9–56.5]	15.4 ± 8.21 95% CI [12.1–18.7]	47.3 ± 26.8 95% CI [36.6–58.0]	t = 0.31 *p* = 0.38 Cd = 0.08	t = 0.43 *p* = 0.33 Cd = 0.12	t = 0.06 *p* = 0.47 Cd = 0.02	t = 0.14 *p* = 0.44 Cd = 0.04
LFHA TENS group (*n* = 25)	16.2 ± 7.41 95% CI [13.3–19.1]	46.8 ± 25.8 95% CI [36.7–56.9]	9.88 ± 6.40 95% CI [7.4–12.4]	31.6 ± 16.9 95% CI [25.0–38.2]	9.5 ± 5.8 95% CI [7.2–11.8]	23.7 ± 13.4 95% CI [18.4–29.0]	t = 3.27 *p* = 0.001 * Cd= 0.91	t = 0.22 *p* = 0.41 Cd = 0.06	t = 2.45 *p* = 0.01 Cd = 0.70	t = 1.81 *p* = 0.04 Cd = 0.52
HFLA TENS group (*n* = 24)	15.8 ± 7.60 95% CI [12.8–18.8]	45.3 ± 23.8 95% CI [35.8–54.8]	13.1 ± 7.51 95% CI [10.1–16.1]	39.7 ± 27.7 95% CI [28.6–50.8]	14.5 ± 8.50 95% CI [11.1–17.9]	41.8 ± 31.1 95% CI [29.4–54.2]	t = 1.25 *p* = 0.11 Cd = 0.36	t = 0.60 *p* = 0.27 Cd = 0.17	t = 0.76 *p* = 0.22 Cd = 0.21	t = 0.25 *p* = 0.40 Cd = 0.07
	Significant differences between LFHA TENS and HFLA TENS groups									
	t = 0.19 *p* = 0.85 Cd = 0.05	t = 0.21 *p* = 0.83 Cd = 0.06	t = 1.62 *p* = 0.11 Cd = 0.46	t = 1.24 *p* = 0.22 Cd = 0.35	t = 2.41 *p* = 0.02 Cd = 0.69	t = 2.66 *p* < 0.01 Cd = 0.76				

Note: EO^1^—eye open before treatment; EO^2^—eye open after treatment; EO^3^—eye open at the end of follow-up; EC^1^—eye closed before treatment; EC^2^—eye closed after treatment; EC^3^—eye closed at the end of follow-up; TENS—transcutaneous electrical nerve stimulation; LFHA—low-frequency high-amplitude; HFLA—high-frequency low-amplitude, t—test statistic, *p*—*p*-value, Cd—Cohen’s d; mean ± standard deviation (SD), confidence interval in mm/second. After application of the Bonferroni correction, the adjusted significance threshold was set at α_adj_ = 0.0028 for intergroup comparisons and α_adj_ = 0.0042 for intragroup dynamics. The asterisk (*) denotes statistically significant differences (*p* < α_adj_).

**Table 5:** Dynamics of tandem walk test across groups.

	Before Treatment		After Treatment		Follow-Up		Significant Difference			
	EO^1^	EC^1^	EO^2^	EC^2^	EO^3^	EC^3^	EO^2^:EO^1^	EO^3^:EO^2^	EC^2^:EC^1^	EC^3^:EC^2^
Control group (*n* = 24)	7.20 ± 1.06 95% CI [6.75–7.65]	3.25 ± 0.89 95% CI [2.87–3.63]	7.37 ± 0.82 95% CI [7.02–7.72]	3.29 ± 0.75 95% CI [2.97–3.61]	7.33 ± 0.96 95% CI [6.92–7.74]	3.41 ± 0.88 95% CI [3.04–3.78]	t = 0.62 *p* = 0.54 Cd = 0.18	t = 0.16 *p* = 0.88 Cd = 0.05	t = 0.17 *p* = 0.87 Cd = 0.05	t = 0.54 *p* = 0.61 Cd = 0.15
LFHA TENS group (*n* = 25)	7.25 ± 1.03 95% CI [6.82–7.68]	3.20 ± 0.58 95% CI [2.96–3.44]	9.70 ± 0.55 95% CI [9.47–9.93]	5.50 ± 0.78 95% CI [5.18–5.82]	9.79 ± 0.41 95% CI [9.62–9.96]	5.75 ± 0.84 95% CI [5.40–6.10]	t = 10.5 *p* < 0.0001 * Cd = 2.97	t = 0.65 *p* = 0.52 Cd = 0.18	t = 11.8 *p* < 0.0001 * Cd = 3.35	t = 1.09 *p* = 0.28 Cd = 0.31
HFLA TENS group (*n* = 24)	7.16 ± 0.96 95% CI [6.75–7.57]	3.16 ± 0.86 95% CI [2.80–3.52]	8.75 ± 1.07 95% CI [8.30–9.20]	4.12 ± 1.11 95% CI [3.65–4.59]	8.83 ± 1.04 95% CI [8.39–9.27]	4.29 ± 1.16 95% CI [3.80–4.78]	t = 5.42 *p* < 0.0001 * Cd = 1.57	t = 0.26 *p* = 0.79 Cd = 0.08	t = 3.35 *p* = 0.002 * Cd = 0.97	t = 0.52 *p* = 0.60 Cd = 0.15
	Significant differences between LFHA TENS and HFLA TENS groups									
	t = 0.30 *p* = 0.76 Cd = 0.09	t = 0.19 *p* = 0.85 Cd = 0.05	t = 3.93 *p* = 0.0008 * Cd = 1.12	t = 5.06 *p* < 0.0001 * Cd = 1.45	t = 4.19 *p* = 0.0003 * Cd = 1.20	t = 4.79 *p* < 0.0001 * Cd = 1.37				

Note: EO^1^—eye open before treatment; EO^2^—eye open after treatment; EO^3^—eye open at the end of follow-up; EC^1^—eye closed before treatment; EC^2^—eye closed after treatment; EC^3^—eye closed at the end of follow-up; TENS—transcutaneous electrical nerve stimulation; LFHA—low-frequency high-amplitude; HFLA—high-frequency low-amplitude, t—test statistic, *p*—*p*-value, Cd—Cohen’s d; mean ± standard deviation (SD), confidence interval in mm^2^. After application of the Bonferroni correction, the adjusted significance threshold was set at α_adj_ = 0.0028 for intergroup comparisons and α_adj_ = 0.0042 for intragroup dynamics. The asterisk (*) denotes statistically significant differences (*p* < α_adj_).

**Table 6:** Dynamics of the modified clinical test of sensory interaction in balance on the foam surface across groups.

	Before Treatment		After Treatment		Follow-Up		Significant Difference			
	EO^1^	EC^1^	EO^2^	EC^2^	EO^3^	EC^3^	EO^2^:EO^1^	EO^3^:EO^2^	EC^2^:EC^1^	EC^3^:EC^2^
Control group (*n* = 17)	25.9 ± 1.80 95% CI [25.04–26.76]	16.5 ± 2.11 95% CI [15.50–17.50]	25.8 ± 1.69 95% CI [25.00–26.60]	16.4 ± 2.18 95% CI [15.36–17.44]	25.6 ± 1.49 95% CI [24.89–26.31]	16.3 ± 2.06 95% CI [15.32–17.28]	t = 0.19 *p* = 0.42 Cd = 0.05	t = 0.43 *p* = 0.33 Cd = 0.12	t = 0.16 *p* = 0.44 Cd = 0.05	t = 0.16 *p* = 0.43 Cd = 0.05
LFHA TENS group (*n* = 18)	25.7 ± 1.62 95% CI [24.95–26.45]	16.4 ± 2.06 95% CI [15.45–17.35]	28.9 ± 1.13 95% CI [28.38–29.42]	21.4 ± 2.69 95% CI [20.16–22.64]	29.0 ± 1.38 95% CI [28.36–29.64]	23.1 ± 2.58 95% CI [21.91–24.29]	t = 8.10 *p* < 0.0001 * Cd = 2.29	t = 0.27 *p* = 0.39 Cd = 0.08	t = 5.62 *p* < 0.0001 * Cd = 1.59	t = 1.08 *p* = 0.14 Cd = 0.64
HFLA TENS group (*n* = 20)	25.6 ± 1.86 95% CI [24.78–26.42]	16.3 ± 1.72 95% CI [15.55–17.05]	28.0 ± 1.55 95% CI [27.32–28.68]	18.2 ± 2.53 95% CI [17.09–19.31]	28.1 ± 1.62 95% CI [27.39–28.81]	18.5 ± 2.98 95% CI [17.19–19.81]	t = 4.95 *p* < 0.0001 * Cd= 1.40	t = 0.22 *p* = 0.41 Cd = 0.07	t = 3.49 *p* = 0.0005 * Cd = 1.0	t = 0.52 *p* = 0.30 Cd = 0.14
	Significant differences between LFHA TENS and HFLA TENS groups									
	t = 0.17 *p* = 0.87 Cd = 0.06	t = 0.16 *p* = 0.87 Cd = 0.05	t = 2.05 *p* = 0.04 Cd = 0.67	t = 3.75 *p* = 0.0006 * Cd = 1.23	t = 2.37 *p* = 0.02 Cd = 0.78	t = 4.88 *p* < 0.0001 * Cd = 1.61				

Note: EO^1^—eye open before treatment; EO^2^—eye open after treatment; EO^3^—eye open at the end of follow-up; EC^1^—eye closed before treatment; EC^2^—eye closed after treatment; EC^3^—eye closed at the end of follow-up; TENS—transcutaneous electrical nerve stimulation; LFHA—low-frequency high-amplitude; HFLA—high-frequency low-amplitude, t—test statistic, *p*—*p*-value, Cd—Cohen’s d; mean ± standard deviation (SD), confidence interval in mm^2^. After application of the Bonferroni correction, the adjusted significance threshold was set at α_adj_ = 0.0028 for intergroup comparisons and α_adj_ = 0.0042 for intragroup dynamics. The asterisk (*) denotes statistically significant differences (*p* < α_adj_).

## Data Availability

Data is unavailable due to privacy and ethical restrictions.

## Supplementary Materials

The following supporting information can be downloaded at: https://www.mdpi.com/article/10.3390/jcm15135000/s1; Table TREND statement checklist [110].
